# Judging residents’ performance: a qualitative study using grounded theory

**DOI:** 10.1186/s12909-018-1446-1

**Published:** 2019-01-08

**Authors:** Marrigje E. Duitsman, Cornelia R. M. G. Fluit, Wieke E. van der Goot, Marianne ten Kate-Booij, Jacqueline de Graaf, Debbie A. D. C. Jaarsma

**Affiliations:** 1 Department of Internal Medicine and Health Academy, Radboud Health Academy, Radboud University Medical Centre, Gerard van Swietenlaan 4, Postbus 9101, 6500 HB Nijmegen, the Netherlands; 2 Health Academy, Department of Research in Learning and Education, Radboud University Medical Centre, Nijmegen, the Netherlands; 3 Martini Hospital, Groningen, the Netherlands; 4 Department of Obstetrics and Gynaecology, Erasmus University Medical Centre, Rotterdam, the Netherlands; 5 Department of Internal Medicine, Radboudumc Nijmegen, Nijmegen, the Netherlands; 6 Centre for Education Development and Research in Health Professions, University Medical Centre Groningen, Groningen, the Netherlands

**Keywords:** Assessment, Postgraduate medical education, Program directors, resident’s performance, Grounded theory

## Abstract

**Background:**

Although program directors judge residents’ performance for summative decisions, little is known about how they do this. This study examined what information program directors use and how they value this information in making a judgment of residents’ performance and what residents think of this process.

**Methods:**

Sixteen semi-structured interviews were held with residents and program directors from different hospitals in the Netherlands in 2015–2016. Participants were recruited from internal medicine, surgery and radiology. Transcripts were analysed using grounded theory methodology. Concepts and themes were identified by iterative constant comparison.

**Results:**

When approaching semi-annual meetings with residents, program directors report primarily gathering information from the following: assessment tools, faculty members and from their own experience with residents. They put more value on faculty’s comments during meetings and in the corridors than on feedback provided in the assessment tools. They are influenced by their own beliefs about learning and education in valuing feedback. Residents are aware that faculty members discuss their performance in meetings, but they believe the assessment tools provide the most important proof to demonstrate their clinical competency.

**Conclusions:**

Residents think that feedback in the assessment tools is the most important proof to demonstrate their performance, whereas program directors scarcely use this feedback to form a judgment about residents’ performance. They rely heavily on remarks of faculty in meetings instead. Therefore, residents’ performance may be better judged in group meetings that are organised to enhance optimal information sharing and decision making about residents’ performance.

**Electronic supplementary material:**

The online version of this article (10.1186/s12909-018-1446-1) contains supplementary material, which is available to authorized users.

## Background

Competency-based medical education (CBME) has been introduced over the past decades to ensure that residents in postgraduate training programmes attain the high standards and competencies that are required to become medical specialists [1–4]. New methods and tools for assessing residents’ clinical competency have been developed to provide residents with feedback to facilitate their progression towards higher levels of performance [5, 6].

To monitor growth in competencies, programmatic assessment has been introduced [7, 8]. Programmatic assessment is based on the idea that using aggregate data from different assessment tools is more reliable and valid to judge residents’ performance than using data from one tool only [9, 10]. Data from multiple formative tools can be combined to make summative judgments of residents’ overall performance [10, 11]. Therefore, residents are expected to collect a selection of different formative assessment tools (for example, mini-CEXs, OSATS) to provide evidence of their competency development [12].

Program directors in postgraduate medical education are responsible for making decisions about residents’ overall performance. They are supposed to do this by aggregating information from various tools, which are completed by different supervisors [13]. In practice it is difficult to aggregate fragmented assessments of different competencies to make a robust decision about residents’ performance [14].

Literature provides a number of relevant insights that can be taken into account when studying how judgments of residents’ performance are established. Oudkerk Pool et al. studied how assessors integrate and interpret different tools from students’ portfolios [15]. Their findings show that assessors find it difficult to judge students without knowing them in person. They felt the need to obtain information about the student’s personality and background. This finding is in line with Whitehead et al. who suggest that, although the psychometric measures in a portfolio are useful and indispensable, they may not be sufficient to judge clinical performance [16]. For an evaluation of performance progress, it is necessary to focus on behavioural objectives, and additionally also on social, personal, affective or ethical learning curves [16–20]. Recent studies show that judgment of residents’ performance is indeed highly influenced by personal and interpersonal characteristics [21, 22]. Supervisors base their assessment of residents on many factors other than skills that are reducible to competency frameworks [21, 23–25], such as personality, motivation, humour and attitude [21].

Little is known about how program directors form judgments of residents’ overall performance and how they feed this back to residents. It is relevant to obtain this knowledge because program directors have the ultimate responsibility for the performance of their residents [3, 13]. The process of how program directors form a judgment on residents’ overall performance is the focus of this paper.

The aims of our study were twofold. The first aim was to investigate what information program directors use and how they value this information in making a judgment of residents’ performance. The second aim was to investigate how residents think that program directors do this, to find out whether there is a gap between what they think is important and what program directors actually value as important. Therefore, we sought to answer the following main research questions: (i) What information do program directors use to make a judgment of residents’ performance and how do program directors value the different sources of information? (ii) What do residents believe about the manner in which program directors make judgements of their performance?

## Method

### Setting

We conducted our study in the Netherlands, where all postgraduate medical training programmes are competency based. In the Netherlands, residents are medical doctors who work under the direct or indirect supervision of medical specialists. They are specialty trainees who are trained in order to obtain a license to practise a chosen specialty. They are trained and supervised by many faculty members, but the program director is the ultimate arbiter of whether progression in residents’ performance is adequate. Program directors must hold evaluation meetings with each resident at least twice a year. They are then expected to give residents an official judgment of their performance (below/at/above expected level) and provide them with sufficient feedback to set goals for further development. Program directors, however, are not necessarily the ones who work together with residents routinely in the clinic. They need to rely on various information sources to know how residents perform at the workplace.

Residents are expected to collect feedback from multiple assessment tools in a portfolio to provide evidence for their progress and competency development. These assessment tools are, for example, results from in-service exams, OSATs and mini-CEXs. The assessment tools contain ratings as well as narrative feedback. The inclusion of the assessment tools in the portfolio is resident-driven with the exception of the in-service exam. Residents decide when to ask a supervisor to complete an assessment form and provide feedback on their performance. Program directors are supposed to make a robust judgment on residents’ progress based on these different data points. Some programmes also hold faculty group meetings to discuss the content of the tools in relation to residents’ performance. These meetings are not mandatory in the Netherlands; program directors can decide whether or not to organize a meeting and if so, how to set up these meetings.

### Data collection and analysis

The goals of this study were to gain insight into the process of how program directors gather information about residents to judge their performance and to explore how residents think about this process. We used a constructive grounded theory approach to do so [26]. Grounded theory is an exploratory research method that seeks to understand the processes that underlie a phenomenon of interest, which makes it a suitable method for our research aim [27]. The grounded theory method needs an iterative process and systematic treatment of data through coding and constant comparisons [28].

Data were collected in 2015–2016. We purposively sampled program directors and residents who were scheduled for a semi-annual evaluation meeting. We recruited participants from internal medicine, radiology and surgery, both from university medical centres and general hospitals. We did not recruit residents from primary care, because their training and assessment program differ from residents in secondary care specialties. We conducted 16 semi-structured interviews with program directors (*N* = 8) and residents (N = 8). The interviews lasted between 45 and 60 min. Program directors differed in years of experience and residents in their year of training (Table 1). We invited program directors and residents by an email invitation. All participants provided written informed consent. We chose to conduct semi-structured interviews with the participants, since we wanted to understand the process of forming a judgment on resident’s performance.

**Table 1:** Participants

	Medical Specialty	Hospital (general hospital / university medical center)	Program Director male/female years of experience as PD	Resident male/female year of training
1	Internal medicine	General hospital	Male 10 years	Male 3rd year
2	Radiology	University medical center	Female 3 years	Male 2nd year
3	Radiology	University medical center	Male 1 year	Male 1st year
4	Internal Medicine	General hospital	Female 2 years	Female 3rd year
5	Internal Medicine	University medical center	Female 1 year	Female 2nd year
6	Radiology	General hospital	Female 5 years	Male 3rd year
7	Surgery	General hospital	Male 8 years	Female 1st year
8	Surgery	University medical center	Male 5 years	Male 4th year

One researcher (MD) conducted all the interviews. Program directors and residents were interviewed separately immediately after they had an evaluation meeting together. Interviews were recorded and transcribed verbatim. All identifying data were omitted. We used two separate but similar interview guides for program directors and residents (see Additional files 1 and 2). We asked program directors what information on residents they used and how they valued this information; we asked residents for their thoughts about this process. Data collection and analysis proceeded in an iterative fashion; they were performed simultaneously, and the processes influenced each other.

Three research team members (MD, WG and CF) separately coded the first four interviews (two interviews with program directors and two with residents), meaning that they organized the data into initial key concepts and themes [28]. After discussing discrepancies in the codes, the three researchers reached agreement about the initial coding list. The initial coding list was discussed in the whole research team and modifications were made. MD and WG analysed the other transcripts, discussed their coding approaches and re-examined earlier transcripts. To inform the coding process, the concepts and themes were periodically discussed in the whole research team. Relations among themes were defined and discussed in the whole team to arrive at a conceptual level of analysis. We stopped data collection when thematic saturation was achieved. Saturation in our study means that we had sufficient data to understand all concepts and themes [28, 29]. The study was approved by the ethical board of the Dutch Association of Medical Education (NVMO) (file number 506).

### Research team and reflexivity

An important principle of constructivist grounded theory is that the construction of concepts and themes arises through interaction with the participants and other researchers in the team [26]. It is important, therefore, to take into account the research team’s background as this influences data interpretation. The lead author (MD) is a medical doctor and worked as a resident in a general hospital; WG has a background in psychology and educational science and works as an educationalist in a general teaching hospital; CF has a medical and educationalist background and is head of a medical education research department; MK is an experienced gynaecologist, program director, and currently the chair of the post-graduate medical education council; JG is a professor of internal medicine, program director, and director of a postgraduate medical education program; and DJ is a professor of medical education with a veterinary background. MD conducted all the interviews and her background inevitably had effect on the study, for example on how the interviews were conducted, which findings were considered most important and how results were interpreted. We tried to mitigate these affects by using a semi-structured interview guide and by ensuring that different perspectives on the data were taken into account; both MD and WG coded all transcripts, they discussed their differences in approaching the coding process and periodically discussed the findings in the entire research team.

## Results

The results of the two main research questions are presented successively. The results are supported by quotes from program directors (O) and residents (A).

### What information do program directors use to make a judgment of residents’ performance and how do they value this information?

We identified three sources of information that program directors predominantly use to form a judgment of the residents’ performance: assessment tools in the portfolio; faculty; and their own experience and personal connection with a resident.

#### The portfolio

O3: There’s a lot of irrelevant information in the portfolio; I have to search for little pieces of relevant information.

Most program directors mainly saw the portfolio as a tool to confirm their judgment already formed by their own experience and remarks made by faculty members. Program directors noticed that the portfolio was almost always filled with good results and positive feedback. If resident’s performance was adequate according to the program director, the portfolio was seen as proof that the resident was indeed performing well. If their judgment on resident’s performance was not in line with what the portfolio’s content appeared to imply, the portfolio was considered an inadequate tool and interpreted as providing an overly positive image of the resident.

One cause of the portfolio being more positive than reality was the fact that faculty seemed hesitant to give negative feedback.

O6: I don’t think the portfolio is always that correct. It mostly says “this is good”, while you may later hear that it wasn’t that good at all and that it was a lot less pretty than the portfolio suggests.

Another reason program directors gave to explain why the portfolio was more positive than reality was that residents often only ask feedback after well-performed tasks and this influences their representation.

O5: The really good residents have many assessment tools filled in their portfolio. They ask for a lot of feedback, also on difficult tasks. But the average ones don’t ask for much feedback, so staff won’t notice that they’re just average residents. They only ask feedback after they performed a task well.

#### Faculty

##### Faculty meetings and comments

Program directors saw faculty meetings as an important source of information about residents because they knew that faculty found it difficult to put negative feedback or points for improvement down on paper, while acknowledging that not all information that was shared in these meetings was useful. There was a tendency to follow the first or the loudest opinion expressed during such meetings. Program directors considered this in the process of judging residents’ performance and took into consideration who said what and how. They were also aware that they put a greater value on some faculty members’ opinions than those of others.

O8: When this person says something, I really see it as a red flag. This person always keeps his opinion to himself and his comment is really astute. When someone else says something, I may think: yeah sure, I hear that three times a week.

##### Group dynamics

Once faculty members had formed an opinion about a resident, they were not prone to change their minds, not even when the resident changed his/her behaviour. Program directors were aware of this and took it into consideration when they valued information about residents. If they noticed that the group followed one member’s opinion or based their opinion on one incident, they did not take it seriously.

O1: I’m annoyed by many of my colleagues. They may have their negative opinion ready in just one second. They don’t say anything for a long time and then suddenly they say “this resident is worth nothing” and they won’t ever change their mind. I don’t like that, you know. But well, my colleagues are all different personalities […] and if I’ve learned anything in the past years as a program director it’s that I know how they judge people. Some of them draw conclusions too soon. I have to take this into account and not take it too seriously. I must be neutral as a program director.

##### Interest in education and training

In general, program directors felt that faculty did not put education and training first. Some faculty members were more interested and involved in teaching and training than others and, as a result, their feedback was more appreciated by program directors.

O1: My personal opinion about my colleagues is important. You know about one person that he’s not interested and doesn’t put any effort in residents’ training. And you know about another person that they take time and think about the feedback they’ll give before they write it down. Yes, that’s why I don’t take everyone seriously.

O3: We have certain subgroups that never complete the assessment tools, or if they do, they do it weeks later. You cannot rely on them. We try to change this, but it’s a persistent problem.

Program directors who felt more supported by their colleagues took their feedback and suggestions about residents seriously:

O2: I don’t teach them [residents], but the whole group does. I think that’s because I give them [faculty] the responsibility: they feel responsible. I couldn’t do it all by myself. I need the interaction with my colleagues; they need to think with me, also about the really good residents.

#### Experience and personal connection with a resident

Program directors explained that their own opinion was an important influence on their overall appraisal. They got to know residents during meetings, nightshifts, and shift-to-shift handovers. Program directors were aware that the personal connection between them influenced their judgment.

O8: There’s something in the assessment process that has to do with the personal bond you feel with this resident, something like a “personal preference”. So there’s a danger that I don’t judge all residents by the same standards.

#### Beliefs

The program directors’ beliefs about teaching and learning seemed to influence how they valued information about residents’ performance and seemed to affect their judgment, as well as the feedback they provided to the residents in the evaluation meeting.

O4: The good residents don’t need much feedback; if all goes well, it can be summed up in one or two sentences, like, erm, “everything goes well, no problems”.

Some program directors expressed their belief that a resident’s level of performance would never change: residents who are not so good will never reach high standards and residents who perform well do not need much feedback.

O5: With a somewhat dysfunctional resident, I don’t think that everything will stay bad, but it’ll never be totally good.

O6: You know immediately how a resident performs. A resident who performs less than the others will never become a really good one.

Other program directors thought that residents’ competencies could grow through training, learning, and applying feedback. They put great value in their judgments on whether or not residents applied constructive feedback and changed their behaviour.

O1: He’s a resident with weaknesses, but he handles critical feedback very well and tries to apply it. I’ve seen him develop and grow in his performance. I value this more than residents who perform well but do nothing with the feedback I provide.

### What do residents think about what information program directors use and how they value this?

#### Portfolio

Residents thought that program directors put great value on the portfolio and they believed they could influence the program director’s assessment through the assessment tools that they collected in their portfolio. They saw these as tools to demonstrate their performance and therefore often only asked supervisors to complete an assessment tool after a well-performed task.

A1: All the good mini-CEXs in my portfolio are a reassurance to me. The program director can do nothing but give me a good appraisal. It is stated in black and white.

A4: As a resident, you can influence your appraisal because if you only put OSCEs on the table after you’ve done something really well, then… well, I mean, what negative things could the program director say about you?

#### Faculty

##### Faculty meetings and comments

Residents knew that faculty talked about them in faculty meetings and in the corridors, but they thought that program directors did not take these comments very seriously, as illustrated by the following quote:

A1: I don’t think they put so much value on these things, especially when it concerns some vague email or some vague comment from a colleague.

Residents were confident that program directors base their judgment on the assessment tools from their portfolios.

##### Interest in education and training

Like program directors, residents noticed that some faculty members do not put teaching and training first. They were critical about the way faculty gave feedback; they felt that their supervisors were hesitant to present points for improvement, did not take enough time to give feedback, and made vague comments on overall performance.

A6: Faculty don’t always think that training residents is interesting or important. They don’t let you get involved in research or they don’t like to teach things. […] I specifically ask for points for improvement, but if the only thing I hear is “keep up the good work”, then I give up. I think to myself “this is hopeless; no matter how many times I ask for constructive feedback, they won’t give it.”

A5: They don’t have the time or don’t like to teach you things. I miss getting feedback. […] I don’t want only positive feedback, but I also want to hear things I can improve. I try to ask for this. I always ask them what they think of my work. I always ask for this. But I miss the spontaneous feedback and I want to learn from the things that didn’t go that well.

##### Group dynamics

Residents felt that once faculty had formed an opinion about them, it was very hard to make them change their minds. Furthermore, they noticed the same group dynamics as program directors did: faculty followed the opinion of the loudest mouths.

A1: When something happens, faculty tend to bear this in mind forever … and I think there’s a minority that talks very loudly. The majority may think differently, but they keep quiet and don’t say a word.

#### Experience and personal connection with a resident

A1: I think that this has been my rescue. I think that having a good relationship with him has really been my rescue.

Residents thought that their personal connection with the program director was of importance and could even alter their appraisal of them.

A4: He [peer resident] has a really good relationship with her [program director]. They just have a really good connection, and he talks his way out of things.

## Discussion

Our findings show that program directors scarcely use feedback from assessment tools to form a judgment on residents’ overall performance, but heavily rely on remarks of faculty in meetings instead. Contrarily, residents think that the feedback in the assessment tools is the most important proof to demonstrate their performance.

The usage of formative assessment tools to facilitate learning, as well as the aggregation of multiple formative assessment tools to come to a summative decision about resident’s performance [11], both seem difficult to actualise in practice. Our results offer various explanations for this difficulty: formative assessment tools are perceived as summative tools, faculty provides poor quality feedback and some program directors think that residents are not able to improve future performance very much. We will discuss these explanations in relation to the literature.

There is a contradiction in what residents wish for and how they act. On one hand, they are disappointed in hardly receiving meaningful feedback, because they feel it does not support their learning process. But on the other hand, residents seem to perceive the assessment tools as summative instead of formative data points. They are under the impression that program directors put much value on the feedback in the assessment tools (both ratings and narrative comments) and seem to think that program directors also perceive the tools as summative assessments. As a result, they only ask feedback after well-performed tasks because they are afraid to receive negative feedback. This is in line with previous research showing that assessment tools are frequently used for summative assessment but not for formative assessment to facilitate learning [30–32].

Moreover, program directors point out that narrative feedback in assessment tools is often vague and predominantly positive on overall performance. This finding is consistent with previous research on feedback in medical education; faculty hesitate to give constructive or negative feedback [33–36]. However, poor quality feedback does not facilitate learning, for residents cannot reflect on vague remarks on global functioning. With this, the profit of collecting different data points comes into question [37, 38].

As a consequence of the above-mentioned issues, program directors cannot rely on feedback in formative assessments to make summative judgments. Instead, they are forced to turn to other sources to get feedback on residents’ performance. They rely on comments of faculty members during faculty meetings. Residents do not know what is discussed in these meetings and they do not receive feedback based on these meetings. At the same time, program directors acknowledge the problem that the information sharing process during faculty meetings is suboptimal. Literature tells us that ineffective information sharing endangers a good group decision and therefore jeopardizes the integrity of the evaluation of residents’ performance [39]. It is therefore important to create conditions in the meetings that ensure effective information sharing.

Furthermore, as the main objective of assessment within CBME is to support residents’ learning and development, it is problematic that some of our interviewed program directors seem to have a fixed-mindset [40] (i.e. they believe residents cannot change and improve their future behaviour much). The literature on self-theories of assessors [41–43] leads us to assume that program directors’ implicit beliefs on learning influence how they value feedback; how they give feedback; and the way they judge residents’ performance. Program directors need to believe that residents can improve their performance by receiving and applying feedback.

### Implications for practice

An obvious recommendation would be to ensure that formative assessment tools are used and perceived as intended and to train program directors, supervisors and residents in asking, giving and receiving meaningful feedback. Research shows however, that despite training, it still remains difficult to bring quality feedback into practice [44–48]. An explanation for this might be the implicit beliefs people have on learning. Addressing these beliefs and creating a developmental belief in a training setting may be worthwhile [40].

Moreover, we recommend that group decision making related to the judgment of residents’ performance (in some derived form of a faculty meeting) becomes obligatory and transparent. In the United Stated, the Accreditation Council for Graduate Medical Education (ACGME) already requires Clinical Competency Committees to determine residents’ competence [49]. To arrive at a good group decision, it is important to create an environment in which good decisions can be made [50]. Crucial in this, is that the meetings must be structured in order to facilitate the best conditions for a good group decision making process, to avoid coming to an agreement too soon and to minimize social influence [50–53]. The ACGME offers a guideline for how to set up these group meetings to create an environment for information sharing and decision making [49]. The meetings should be transparent for residents and they should receive feedback based on these meetings, as to further stimulate their learning and development.

### Strengths and limitations

A strength of our study is the diversity of our sample: we included participants from different hospitals, medical specialties, and experience levels in training and teaching. A limitation of our study is that we used a rather small sample in the specific postgraduate medical context of the Netherlands and we do not know if our results would apply to other program directors, programs or other countries.

We believe, though, that our study lays a foundation for future research in other settings, so to further our understanding on how to optimize the process of forming robust and acceptable judgment on residents’ performance progress.

## Conclusion

Residents think that the feedback in the assessment tools is the most important proof to demonstrate their performance, whereas program directors scarcely use this feedback to form a judgment about residents’ performance. The objective of aggregating formative assessment tools to form a summative judgment is difficult to reach in practice. Formative assessment is perceived as being summative and faculty provides poor quality feedback. Program directors rely heavily on remarks of faculty in meetings instead. They acknowledge that there is no optimal environment for good decision making during these meetings. We suggest that group decision making concerning residents’ performance becomes obligatory, provided that these meetings are set up according to guidelines that support an environment for optimal information sharing and decision making. Furthermore, they should accommodate high quality feedback to residents, in order to facilitate their learning.

## Additional files

Additional file 1: Appendix 1 Semi-structured interview guide program director. The semi-structured interview guide we used for the interviews. (DOCX 54 kb)

Additional file 2: Appendix 2 Semi-structured interview guide resident. The semi-structured interview guide we used for the interviews with the residents. (DOCX 56 kb)

## Abbreviations

ACGME: Accreditation Council for Graduate Medical Education
CMBE: Competency Based Medical Education
mini-CEXs: mini-Clinical Evaluation Exercise
OSATS: Objective Structured Assessment of Technical Skills
