# Supplementary material for: Judging residents’ performance: a qualitative study using grounded theory
Source: BMC Med Educ. 2019 Jan 8;19:13. doi: 10.1186/s12909-018-1446-1 (PMC6325830; doi:10.1186/s12909-018-1446-1)
Supplement: Supplementary file 1 — Appendix 1 Semi-structured interview guide program director. The semi-structured interview guide we used for the interviews. (DOCX 54 kb) [file 12909_2018_1446_MOESM1_ESM.docx]

**Appendix 1**

**Semi-structured interview guide**

**Program director**

What are your thoughts about the evaluation meeting that just took place?

Probes:

Did the meeting go according to plan/expectation?

Did something happen/was something said that surprised you?

**Preparation**

How did you prepare for this meeting?

What information did you use to make a holistic evaluation of the resident’s competence?

Where did you find or hear this information?

How do you value this information?

Probes:

What is the most important information for you?

Can you explain why this is the most important?

**The evaluation meeting**

What information did you share with the resident?

Why did you choose to share this information?

Probes:

I heard that [this] was talked about in the meeting. Why did you choose to bring this up? Are there other things that where important in making your evaluation of the resident? Why did you choose not to explicitly talk about this?

What did you want this resident to learn from this meeting?

**General:**

Why did you use [these instruments] to make your evaluation of the resident?

Why don’t you use [other instruments]?

Do you miss something in the residents’ portfolios?

When do you know (during the training program) at what level a resident performs?

What do you think is important to be a good resident?

What do you think faculty believe is important to be a good resident?
