# Supplementary material for: Judging residents’ performance: a qualitative study using grounded theory
Source: BMC Med Educ. 2019 Jan 8;19:13. doi: 10.1186/s12909-018-1446-1 (PMC6325830; doi:10.1186/s12909-018-1446-1)
Supplement: Supplementary file 2 — Appendix 2 Semi-structured interview guide resident. The semi-structured interview guide we used for the interviews with the residents. (DOCX 56 kb) [file 12909_2018_1446_MOESM2_ESM.docx]

**Appendix 2**

**Semi-structured interview guide**

**Resident**

What do you think about the evaluation meeting that just took place?

Probes:

Did the meeting go according to plan/expectation?

Did something happen/was something said that surprised you?

**Preparation**

How do you think the program director prepared for this meeting?

What information do you think he/she uses to make a holistic evaluation of your competence?

Where do you think he/she found or heard this information?

How do you think he/she values this information?

Probes:

What do you think is the most important information for the program director?

Can you explain why this is the most important?

**The evaluation meeting**

What information did the program director share with you during the meeting?

Why do you think he/she chose to share this information?

Probes:

I heard that [this] was talked about in the meeting. Why do you think the program director chose to bring this up? Do you think that there are other important things that he/she uses to evaluate your performance?

What did you learn from this meeting?

**General:**

How can you show as a resident that you have developed your competencies?

What instruments do you think are important for a program director to evaluate your performance?

Why do you think these are important?

Do you miss something in the portfolio?

What do you think is important to be a good resident?

What do you think a program director finds important to be a good resident?

What do you think that faculty finds important to be a good resident?
